# Retention of functional variation despite extreme genomic erosion: MHC allelic repertoires in the *Lynx* genus

**DOI:** 10.1186/s12862-017-1006-z

**Published:** 2017-07-04

**Authors:** Elena Marmesat, Krzysztof Schmidt, Alexander P. Saveljev, Ivan V. Seryodkin, José A. Godoy

**Affiliations:** 1 Department of Integrative Ecology, Estación Biológica de Doñana (CSIC), C/Américo Vespucio, 26, 41092 Sevilla, Spain; 2 Mammal Research Institute, Polish Academy of Sciences, 17-230 Białowieża, Poland; 3 Department of Animal Ecology, Russian Research Institute of Game Management and Fur Farming, 79 Preobrazhenskaya Str, Kirov, 610000 Russia; 4 Laboratory of Ecology and Conservation of Animals, Pacific Institute of Geography of Far East Branch of Russian Academy of Sciences, 7 Radio Street, Vladivostok, 690041 Russia; 5 Far Eastern Federal University, 8 Sukhanova Street, Vladivostok, 690091 Russia

**Keywords:** Mhc, Lynx, Balancing selection, Natural selection, Genetic functional diversity, Bottleneck, Drift, Iberian lynx, Conservation genetics, Recombination

## Abstract

**Background:**

Demographic bottlenecks erode genetic diversity and may increase endangered species’ extinction risk via decreased fitness and adaptive potential. The genetic status of species is generally assessed using neutral markers, whose dynamic can differ from that of functional variation due to selection. The MHC is a multigene family described as the most important genetic component of the mammalian immune system, with broad implications in ecology and evolution. The genus *Lynx* includes four species differing immensely in demographic history and population size, which provides a suitable model to study the genetic consequences of demographic declines: the Iberian lynx being an extremely bottlenecked species and the three remaining ones representing common and widely distributed species. We compared variation in the most variable exon of the MHCI and MHCII-DRB loci among the four species of the *Lynx* genus.

**Results:**

The Iberian lynx was characterised by lower number of MHC alleles than its sister species (the Eurasian lynx). However, it maintained most of the functional genetic variation at MHC loci present in the remaining and genetically healthier lynx species at all nucleotide, amino acid, and supertype levels.

**Conclusions:**

Species-wide functional genetic diversity can be maintained even in the face of severe population bottlenecks, which caused devastating whole genome genetic erosion. This could be the consequence of divergent alleles being retained across paralogous loci, an outcome that, in the face of frequent gene conversion, may have been favoured by balancing selection.

**Electronic supplementary material:**

The online version of this article (doi:10.1186/s12862-017-1006-z) contains supplementary material, which is available to authorized users.

## Background

It is crucial for conservation and evolutionary biology to understand how population bottlenecks shape genetic diversity, as its loss might increase extinction risk via decreased reproduction, survival and adaptive potential [1]. Genetic variation is commonly assessed using neutral markers like microsatellites, which have proven useful to infer demography, population structure, and kinship, among other applications. However, neutral markers by definition are not subjected to natural selection, so we cannot assume they adequately reflect the dynamics of functional genetic variation that is most relevant for species persistence (e.g., [2, 3]). We thus need to focus on functionally relevant loci to fully understand how population declines may increase extinction risk through their effects on genetic variation [4–7]. A high concern in conservation, which is influenced by such functional genetic variation, is the species capacity to immunologically respond to pathogens, because it may largely determine their survival and long term viability [8, 9].

The Major Histocompatibility Complex (MHC) is a multigene family often described as the most important genetic component of the mammalian immune system [10]. The MHC encodes cell surface glycoproteins that bind antigens derived from pathogens or parasites and present them to T-lymphocytes, triggering the adaptive immune response. There are two major types of MHC gene families: MHC class I and MHC class II. MHC class I genes are expressed on the cell membrane of all nucleated cells and defend against intracellular threats (as viral pathogens) and malignant cells. MHC class II genes are mainly expressed on specialized antigen-presenting cells of the immune system, which monitor the extracellular environment and use MHC receptors to present peptides of extracellular pathogens to the T-cells.

Previous studies on MHC have evidenced the long-term operation of natural selection (reviewed in [11]) and highlighted its important implications in evolutionary ecology and evolution (reviewed in [12]). It predisposes the MHC as a model for studying the non-neutral genetic evolution in non-model species.

The MHC genes are expected to evolve through natural selection in response to the selection pressures imposed by pathogens. The nature of the molecule, that interacts both with the antigens (i.e., the Antigen Binding Sites, onwards ABS) and the cellular and immune system components, makes it subject to different types of natural selection. On one hand, positive selection signals are expected in the ABS as they may evolve to bind novel antigens carried by pathogens, which in turn may evolve to escape recognition. On the other hand, purifying selection will act on non-ABS regions where most non-synonymous substitutions are likely to be deleterious. Additionally, MHC genes are one of the best characterized targets of balancing selection, which maintain high levels of polymorphisms by either overdominance, rare-allele advantage, fluctuating selection and divergent allele selection [13, 14]. All these forms of evolution are not mutually exclusive, on the contrary, they are likely to act at the same time and interact with each other. For example, divergent allele selection is expected to boost the effect of overdominance and rare-allele advantage [14]. Evidence for long-term balancing selection includes the occurrence of trans-species polymorphisms (TSP), i.e., alleles that are more similar between related species than alleles within each species and this similarity is not due to convergent evolution. TSP is generated by the passage of alleles from ancestral to descendant species due to increased coalescence times in the loci target of balancing selection. Another signature of balancing selection is the excess of non-synonymous polymorphisms, especially at ABS [15]. Since the net result of balancing selection is that it maintains polymorphisms, it is a matter of debate in the context of species conservation whether it can hamper the loss of genetic diversity driven by genetic drift and delay the fixation of alleles. The empirical evidence for this is conflicting, with some MHC studies finding high MHC diversity in otherwise genetically eroded populations (e.g., [2, 16–18]), and some others finding the opposite (e.g., [19–22]).

The MHC diversity and TSP can be studied at three different levels. First, the nucleotide level which is the lower and more stringent one. Second, the protein level in which slightly different nucleotide sequences are functionally the same as they translate into the same protein sequence. Third, the supertype level, that which groups functionally similar alleles into the same supertypes. MHC alleles defined by nucleotide or even amino acid differences may be functionally similar if they bind similar repertoire of antigens. Hence, Functionally redundant alleles can be identified and grouped in the same supertype based on their biochemical similarities at amino acids known to interact with the antigen (i.e., ABS) [23].

In felids MHC genes have received special attention due to the role of domestic cat as a model for human diseases [24–27] and because of its possible impact on endangered wild felid conservation, including the paradigmatic case of the cheetah [18, 28–32].

**Fig. 1:**
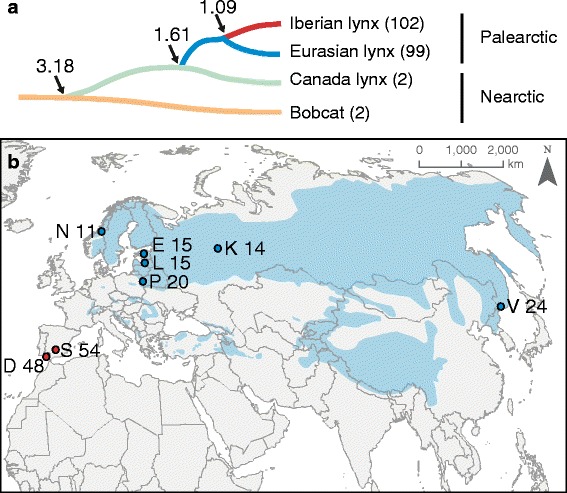
Phylogenetic and geographic scope of the study. **a** Phylogeny of the *Lynx* genus (modified from [35]. The divergence time in MY is showed for each split as well as the sample size per species (in brackets). **b** Geographic distribution of the Palearctic lynxes and populations sampled. For the Iberian lynx the two remnant populations were sampled while the Eurasian lynx was sampled throughout Eurasia. *Shaded* areas indicate the species distribution according to the UICN (http://maps.iucnredlist.org/map.html?id=12519). Each population is coded with a single letter (D = Doñana and S = Sierra Morena for the Iberian lynx and N = Norway, E = Estonia, L = Latvia, P = Poland, K = Kirov, and V = Vladivostok for the Eurasian lynx) along with the number of individuals sampled from each population

The genus *Lynx* being composed of four species: the bobcat (*L. rufus*), the Canada lynx (*L. canadensis*), the Eurasian lynx *(L. lynx*) and the Iberian lynx (*L. pardinus*) ([33, 34]; Fig. 1a) provide a proper model on which to assess the genetic consequences of demographic decline and fragmentation on the functional genetic variation. The four species differ immensely in recent demographic history, population size, and genetic status as well as their phylogenetic relationships. In our model the Iberian lynx represents the highly bottlenecked species and the other three are common and widely distributed species. The Iberian lynx and the Eurasian lynx are sister species, both sharing their most recent common ancestor around 1,5–1.09 Mya, as estimated by phylogenomic methods ([33, 34], Fig. 1a), whereas coalescent methods applied to the whole-genome sequences suggest a more recent divergence (312.2 kya; 95% CI: 323.1–179.4 kya), followed by a long period of gene flow that ceased recently (2.473 kya; 95% CI: 126.8–0 kya) [35].

The Iberian lynx was recognized as the most endangered felid in the world, due to a dramatic decline of its entire population during the last two decades of twentieth century from above 1000 to 100 individuals [36, 37]. In sharp contrast to the Iberian, the Eurasian lynx shows one of the widest distributions among felids, covering a high variety of environmental, ecological, climatic and demographic conditions [38]. The current distribution of the Eurasian lynx extends across Eurasia with the core of its population in Russia. However, in Europe, the Eurasian lynx has also gone through a recent decline that has resulted in its extirpation from most of western Europe and the fragmentation of its westernmost range [39–41]. The bobcat and the Canada lynx are distributed across North America in southern and northern latitudes, respectively, with areas of range overlap in northern United States and southern Canada.

Concordant with the demographic history of the species the Iberian lynx neutral genetic variability is extremely reduced [42], its current genome- and species-wide diversity being the lowest ever reported for any species [35]. In contrast, the bobcat, Canada, and Eurasian lynx, based on mtDNA and microsatellites have shown moderate levels of genetic diversity and little genetic structure across most of their ranges [39, 43, 44]. Hence, the *Lynx* genus arises as a good model to study patterns of functional genetic variation following extreme overall genetic erosion.

Here we characterize the allele repertoire of the most variable exon (exon 2) of MHC class I and class II-DRB genes in the Lynx genus, and test whether a genetically eroded species can maintain a similar range of functional diversity to demographically and genetically healthy related species. More specifically, we tackle the following questions: i) What are the levels of diversity at MHC class I and class II-DRB loci in the Lynx genus?, ii) are there signatures of positive or purifying selection in lynx MHC alleles?, iii) How are the different lynx alleles related among them and to those in other felids; what is the level of TSP across species?, iv) how does the allele repertoire translate into functional diversity in the form of supertypes?, and v) how much of this functional diversity is maintained in Iberian lynx compared to the genetically healthier relatives?

## Results

### Data processing and allele validation

We obtained a total of 554,478 reads with a mean coverage of 2670 (range: 9–16,729) and 563 (range: 12–8964) for MHCI and MHCII-DRB per replicates respectively. Only replicates with coverages above 500 reads for MHCI and 100 for MHCII-DRB were taken into account. Within the *Lynx* genus we found a total of 37 MHCI alleles and 13 MHCII-DRB alleles (KY769287-KY769350 and KY769351-KY769367 respectively, for sequence alignments see Additional file 2: Tables S1 and S2). The number of alleles found within individuals ranged from 10 to 16 for MHCI and from 3 to 6 for MHCII-DRB suggesting a minimum of 5 and 3 amplified loci, respectively. Six of the *L. lynx* MHCII-DRB alleles were previously found on Chinese Eurasian lynxes (Lyly-DRB*1, *2, *3, *7, *8 and *10) [29]. The alleles not found in our samples but found in Wang et al. [29] were not included in the analysis, as we considered that the use of a different genotyping methodology (both in the wet lab and filtering steps) could bias the diversity of Eurasian lynx compared with the other lynxes. Nevertheless, we included them in the analysis comparing the lynx alleles sequences with published alleles of Felidae, as most of the published sequences also differ in how they were generated.

None of the MHCII-DRB alleles showed signals of pseudogenization. Therefore, all of them were considered as functional alleles in downstream analyses. On the other hand, seven MHCI alleles presented pseudogenization signals and were excluded from diversity, recombination, selection and supertype analyses. Three of these alleles included deletions, another three insertions (two of them causing a stop codon to appear within the sequenced exon) and the last one harboured a premature stop codon but no indels. They were found across all lynx species (3 in *L. pardinus*, 3 in *L. lynx*, 2 in *L. canadensis* and 5 in *L. rufus* for) and the pattern of sharedness was similar to that found in functional ones (further details in Additional file 1: Table S3).

### Recombination and phylogenetic relationships of the alleles

Both MHC genes showed signals of recombination. For the MHCI sequences SBP inferred recombination with 100% support and suggested position 201 as the recombination breakpoint, whereas GARD lacked power to infer recombination. SBP also inferred recombination in MHCII-DRB sequences and indicated nucleotide position 114 as a recombination breakpoint; GARD confirmed such breakpoint and pointed to an additional one at nucleotide 209. Recombination events contribute to the complexity of the phylogenetic networks (Additional file 2: Figures S1 and S2), with multiple connection links between alleles. The phylogenetic networks also show little structuring of MHC alleles across species, with most clades containing alleles of all lynx species.

**Fig. 2:**
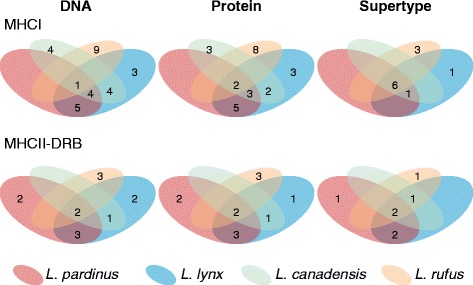
Trans-species polymorphism at MHCI and MHCII-DRB in the *Lynx* genus. The number of shared nucleotide and protein sequences and functional types (supertypes) between lynx species is shown

**Fig. 3:**
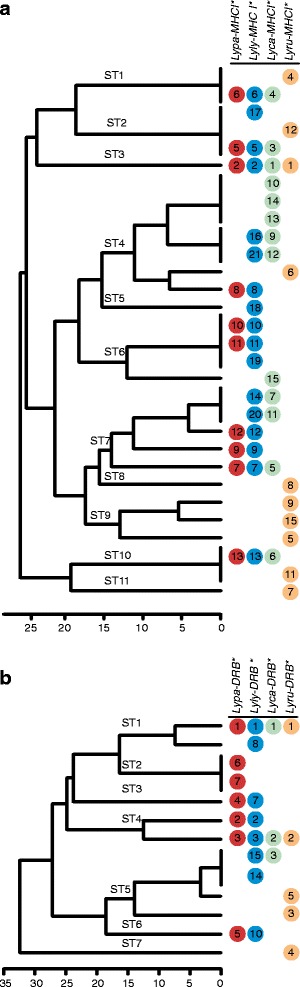
Lynx MHC class I and class II DRB supertypes. We used average hierarchical clustering and a cut-off threshold of Euclidean distance ≥15 to classify alleles into distinctive functional groups (supertypes, ST) based on functional similarity of their ABS. Alleles with identical amino acids at ABS are represented as polytomies at 0 Euclidean distance. Alleles in the same line are identical at the nucleotide level. Alleles are specified with their allele number in each species

We found many alleles to be shared among species within the genus (Fig. 3; nb, even though Fig. 3 focus on supertypes description nucleotide and amino acid differences are also depicted). The percentage of alleles within each species found in any other species was: 100% in *L. pardinus*, 82% in *L. lynx*, 69% in *L. canadensis* and 10% in *L. rufus* for MHCI, and 71% in *L. pardinus*, 75% in *L. lynx*, 100% in *L. canadensis* and 40% in *L. rufus* for MHCII-DRB (Figs. 2 and 3). Shared alleles were less frequent in *L. rufus*, as expected by its basal position in the phylogeny and its older separation from the rest (3.48 MYA versus the *L. canadensis* split which occurred 1.61 MYA) [34]. In contrast, the trio *L. canadensis*, *L. lynx* and *L. pardinus* showed similar high levels of shared polymorphisms despite *L. canadensis* splitting from the Palearctic species 0.52 MY before *L. lynx* and *L. pardinus* separated. The only two MHC alleles that are private of *L. pardinus* are further discussed below (see Functional diversity retention section).

### Signatures of selection

To search for signatures of long-term positive and purifying selection we compared the rates of nonsynonymous (d_N_) and synonymous (d_S_) substitutions for ABS and non-ABS (Table 1), which were congruent across the genus. None of the MHC gene families studied showed d_N_/d_S_ ratios above one (one of the signals of positive selection) neither at ABS nor at non-ABS. On the contrary, they showed purifying selection signals (i.e., d_N_ and d_S_ ratios below one). At the MHCI exon 2 a slight signature of purifying selection was detected at the ABS of *L. canadensis* and *L. pardinus* and at the non-ABS of *L. canadensis* and *L. lynx*. On the other hand, MHCII-DRB exon 2 showed strong signals of purifying selection only at the non-ABS for all species but *L. canadensis*.

**Table 1:** Nonsynonymous (d_N_) and synonymous (d_S_) substitutions (± standard error) and their ratio in ABS and non-ABS for MHCI and MHCII-DRB sequences

	ABS				non-ABS				ABS vs non-ABS	
Species	d_N_	d_S_	d_N_/d_S_	*P*	d_N_	d_S_	d_N_/d_S_	*P*	d_S.ABS_/d_S.non-ABS_	*P*
MHCI (17/59)										
Total	0.26 ± 0.022	0.6 ± 1.39	0.43	n.s	0.06 ± 0.001	0.08 ± 0	0.73	***	7.706	***
*L. pardinus*	0.3 ± 0.027	0.68 ± 2.35	0.44	*	0.07 ± 0.001	0.1 ± 0.01	0.76	n.s	6.972	***
*L. lynx*	0.26 ± 0.022	0.43 ± 1.36	0.59	n.s	0.06 ± 0.001	0.08 ± 0	0.73	*	5.443	***
*L. canadensis*	0.28 ± 0.024	0.68 ± 1.23	0.4	**	0.06 ± 0.001	0.09 ± 0	0.67	**	7.466	***
*L. rufus*	0.33 ± 0.032	1.01 ± 1.55	0.33	n.s	0.08 ± 0.002	0.1 ± 0.01	0.81	n.s	9.89	***
MHCII-DRB (24/58)										
Total	0.19 ± 0.011	0.17 ± 0.03	1.09	n.s	0.06 ± 0.001	0.13 ± 0.01	0.46	***	1.354	**
*L. pardinus*	0.14 ± 0.008	0.16 ± 0.03	0.88	n.s	0.06 ± 0.001	0.14 ± 0.01	0.4	***	1.166	n.s
*L. lynx*	0.16 ± 0.009	0.15 ± 0.02	1.1	n.s	0.06 ± 0.001	0.14 ± 0.01	0.43	***	1.057	n.s
*L. canadensis*	0.19 ± 0.011	0.2 ± 0.03	0.99	n.s	0.07 ± 0.002	0.16 ± 0.01	0.48	n.s	1.251	n.s
*L. rufus*	0.26 ± 0.016	0.23 ± 0.03	1.11	n.s	0.07 ± 0.001	0.12 ± 0.01	0.59	*	1.907	**

The number of amino acids in each typology appears in brackets (ABS/non-ABS). d_N_ is the number of nonsynonymous substitutions and d_S_ the number of synonymous substitutions per site category (ABS or Non-ABS). P is the probability (using a Mann-Whitney U-test) that d_N_ and d_S_ are different at each site type or that d_S_ differ between ABS and Non-ABS

Even though we did not find positive selection at ABS in the form of an elevated d_N_/d_S_ ratio), we found that for MHCI d_S_ at the ABS were from five to ten times higher than d_S_ at the non-ABS. This indicates an older origin of ABS regions when compared to non-ABS, what has been interpreted as a signal of gene-conversion coupled with positive selection [45].

Regarding the site-by-site set of analyses, we found SLAC test to lack power to detect selected sites on our small datasets and REL to suffer from a high rate of false positives- probably because our alignment is too short [46]. Interestingly, we found a good correlation between FEL and FURBAR results. As such analyses are the most suitable for intermediate size datasets, we considered under selection those sites identified by both tests or those identified in one and with borderline values on the other. For MHCI only one amino acid showed signals of positive selection, and ten of negative selection (Additional file 1: Tables S4 and S5). Similarly, for MHCII-DRB the signals of negative (six amino acids) were more pervasive than of positive selection (one amino acid) (Additional file 1: Tables S6 and S7). As expected, we found all negatively selected codons to be non-ABS and the positively selected site in MHCII-DRB to be an ABS. However, the positively selected site in MHCI was not an ABS.

### Functional diversity retention: Genetic diversity and supertypes

The alignment of lynx MHCI (229 bp) and MHCII-DRB (247 bp) nucleotide sequences revealed 86 and 45 variable sites, respectively (Additional file 1: Tables S1 and S2). At the protein level, the sequenced MHCI α_1_ domain (i.e., the translation of the MHCI exon 2) consisted of 76 residues (positions 8–83; Additional file 1: Table S5) with 45 segregating sites, and the sequenced MHCII-DRB ß_1_ domain (i.e., the translation of the MHCII-DRB exon 2) consisted of 82 amino acids (positions 9–90; Additional file 1: Table S7) with 28 variable sites. In both cases, 14 of the variable sites were ABS. Genetic distance patterns along the molecule were similar for the four lynx species (Table 2). The overall mean genetic distance was ~0.1 for both gene classes at nucleotide sequences, whereas it increased to ~0.2 when considering amino acid distances. Moreover, the mean nucleotide and amino acid distances were much higher in the ABS than in the non-ABS, for both gene classes and all four species, indicating that genetic variation at the studied exons concentrate at ABS, as expected.

**Table 2:** Genetic diversity in MHCI and MHCII-DRB in the *Lynx* genus. Diversity at the nucleotide, amino acid and supertype levels are estimated as the number of observed alleles (allelic richness, AR), number (S) and proportion of segregating sites [S(%)], mean number (± standard error) of differences among alleles (K). The latter is calculated for all sites, ABS and non-ABS

Indice	Total	*L. pardinus*	*L. lynx*	*L. canadensis*	*L. rufus*
MHC Class					
*Diversity level*					
MHC Class I					
*DNA (299)*					
AR	30	10	17	13	10
Private		0	3	4	9
S	86	72	73	76	76
S (%)	37.55	31.44	31.88	33.19	33.19
K	19.77 ± 0.52	24.11 ± 1.74	20.07 ± 1	21.51 ± 1.42	25.8 ± 1.64
Gen. dist. All	0.094 ± 0.003	0.116 ± 0.009	0.096 ± 0.005	0.103 ± 0.007	0.125 ± 0.009
Gen. dist. ABS	0.23 ± 0.01	0.27 ± 0.02	0.23 ± 0.01	0.24 ± 0.02	0.29 ± 0.02
Gen. dist. Non-ABS	0.06 ± 0	0.08 ± 0.01	0.06 ± 0	0.07 ± 0.01	0.08 ± 0.01
*Protein (76)*					
AR	26	10	15	10	10
Private		0	3	3	8
S	45	38	39	39	40
S (%)	59.21	50	51.32	51.32	52.63
K	16.68 ± 0.4	19.97 ± 1.36	16.78 ± 0.79	17.27 ± 1.16	21.93 ± 1.28
Gen. dist. All	0.189 ± 0.005	0.231 ± 0.018	0.191 ± 0.01	0.199 ± 0.015	0.256 ± 0.017
Gen. dist. ABS	0.53 ± 0.01	0.58 ± 0.04	0.5 ± 0.02	0.54 ± 0.04	0.68 ± 0.04
Gen. dist. Non-ABS	0.11 ± 0	0.15 ± 0.01	0.12 ± 0.01	0.13 ± 0.01	0.17 ± 0.01
*Supertypes*					
Number	11	7	8	7	8
Private		0	1	0	3
MHC Class II-DRB					
*DNA (247)*					
AR	13	7	8	3	5
Private		2	2	0	3
S	61	47	49	41	56
S (%)	24.7	19.03	19.84	16.6	22.67
K	23.71 ± 1.01	22.33 ± 1.81	23.39 ± 1.76	27.67 ± 4.37	29.1 ± 2.13
Gen. dist. All	0.104 ± 0.005	0.097 ± 0.008	0.103 ± 0.008	0.122 ± 0.021	0.129 ± 0.01
Gen. dist. ABS	0.19 ± 0.01	0.15 ± 0.01	0.16 ± 0.01	0.2 ± 0.04	0.26 ± 0.03
Gen. dist. Non-ABS	0.07 ± 0	0.07 ± 0.01	0.08 ± 0.01	0.09 ± 0.01	0.08 ± 0.01
*Protein (82)*					
AR	12	7	7	3	5
Private		2	1	0	3
S	28	23	23	23	27
S (%)	34.15	28.05	28.05	28.05	32.93
K	16.17 ± 0.7	14.63 ± 1.23	15.72 ± 1.25	19.51 ± 3.73	20 ± 1.51
Gen. dist. All	0.18 ± 0.008	0.161 ± 0.014	0.175 ± 0.015	0.221 ± 0.046	0.226 ± 0.018
Gen. dist. ABS	0.38 ± 0.02	0.31 ± 0.03	0.36 ± 0.04	0.46 ± 0.15	0.5 ± 0.06
Gen. dist. Non-ABS	0.11 ± 0.01	0.11 ± 0.01	0.11 ± 0.01	0.14 ± 0.02	0.14 ± 0.01
*Supertypes*					
Number	7	5	5	3	4
Private		1	0	0	1

Admittedly, we underestimated diversity indices for Nearctic lynxes due to our limited sampling, so we focus on the two Palearctic species for interspecies comparisons. The allelic richness of *L. pardinus* for MHCI exon 2 was by 41% lower than in its sister species *L. lynx* (10 and 17, respectively; Table 2). A similar pattern was found at the protein level, with 10 alleles in *L. pardinus* and 15 in *L. lynx*. However, the number of variable sites at both levels were similar- with 72 and 73 at MHCI exon 2, and 38 and 39 at MHCI α_1_ domain, for Iberian and Eurasian lynx, respectively. Hence, the mean number of pairwise differences between MHCI alleles was higher for *L. pardinus* than for *L. lynx*, both at the protein (19.97 ± 1.36 > 16.78 ± 0.79) and the nucleotide levels (24.11 ± 1.74 > 20.07 ± 1.00). This pattern points to divergent allele selection as the retained subset of alleles retains most of the variable sites, despite being nearly half of the original one. The two Palearctic species showed a similar number of alleles and variable sites for MHCII-DRB exon 2 nucleotide sequences (AR = 7 and 8 and S = 47 and 49) and identical numbers in protein sequences (AR = 7 and S = 23). Therefore, the mean number of differing positions was similar for both levels (Table 2). The MHCII-DRB diversity observed in *L. pardinus* was comparable to that of *L. lynx* in all the indices calculated; indicating a remarkable retention of MHCII-DRB alleles in the former despite its extreme overall genomic erosion [35].

The MHC alleles were grouped into distinctive functional groups (supertypes) according to physicochemical similarities at ABS and using an arbitrary threshold Euclidean distance of ≥15 (Fig. 3). We found 11 MHCI supertypes: five shared among the four species in the genus, two shared by all species but *L. rufus*, three exclusive to *L. rufus* and another one exclusive to *L. lynx*. In the MHCII-DRB case we found 7 supertypes: two shared among the four lynx species, another two shared only among the Palearctic ones, one shared by all but *L. pardinus*, one exclusive to *L. rufus* and another one to *L. pardinus*. The total number of supertypes (MHCI/MHCII-DRB) by species was *L. pardinus* (7/5), *L. lynx* (8/5), *L. canadensis* (7/3), *L. rufus* (8/4).

The only example of alleles private to Iberian lynx were Lypa-DRB*6 and Lypa-DRB*7, which differ only by one base from each other (Figs. 2 and 3). This pair of alleles only add one segregating site to the alignment (the one differentiating Lypa-DRB*6 from Lypa-DRB*7) and can be explained as the result of a single recombination event between some pairs of the other extant alleles. The required recombination point is located between positions 92 and 145, within the range of sites identified in the recombination site analysis. These Iberian lynx private alleles form a separate clade in the phylogenetic networks and a unique supertype cluster.

## Discussion

Here we provide evidence that species-wide functional genetic diversity can be maintained even in the face of severe population bottlenecks which caused devastating whole genome genetic erosion. Our results suggest that this could be the consequence of divergent alleles being retained across paralogous loci, an outcome that, in the face of frequent gene conversion, may have been favoured by balancing selection.

We characterized MHC variation in a quite extensive sampling of *L. pardinus* and *L. lynx* comprehending most of the species range. All previous characterization of MHC variation in lynx and other felids have used PCR with degenerate primers, followed by cloning and Sanger sequencing or SSCP, methods that are probably affected by amplification bias and other artefacts [47, 48]. Our application of improved amplification strategies, high-throughput sequencing approaches and efficient validation algorithms should minimize these problems. The combination of improved genotyping protocols and extensive Palearctic species sampling should have resulted in a rather complete and artefact-free dataset. Even though our sampling of the Nearctic species was poor, the inclusion of these two species enabled a genus-wide evaluation of genetic variation at MHC loci. To the best of our knowledge, this is the first time that the MHC variation of all the species of a genus in the Felidae family has been characterized.

We also believe that we have made a reasonably complete sampling of paralogs in each MHC family. The number of MHC loci described of the Iberian lynx genome annotation [35] is compatible with the number of alleles we found per individual in all lynx species (i.e., MHCI: 10 annotated paralogs and 10–13 alleles per individual and MHCII-DRB: 3 paralogs and 3–6 alleles per individual). Admittedly, the genome assembly could have collapsed some paralogs into one or split some others. However, we do not think this is very likely as many of the paralogs were assembled from different fosmid pools [35]. Moreover, these numbers of paralogs is similar to the numbers found in domestic cat, where the genomic region containing the MHC genes was characterized in detail (MHCI: 12 functional paralogs and MHCII-DRB: 3 functional paralogs) [27], and in other felids; e.g. three paralogs were also reported for the MHCII-DRB in cheetah and leopard [30, 31]. Surprisingly, Wang et al. [29] reported from 1 to 6 alleles per *L. lynx* individual in MHCII-DRB suggesting allelic dropout or copy number variation. However, by applying improved amplification and validation methods we found no evidence of copy number variation at MHCII-DRB in any lynx species, as we consistently found from 3 to 6 alleles per individual. Lower than three allele per individual is thus most likely the result of alleles being missed by their genotyping approach based on degenerate primer amplification, cloning and Sanger sequencing [47, 49].

### Patterns of TSP and their possible origin

The four species in the *Lynx* genus showed a high level of shared MHC polymorphisms across species, a feature in common with other recently diverged taxa [28, 29, 50, 51]. As Wei et al. [28] found for several felids in MHCII-DRB, we also detected instances of TSP between lynxes and other distant felids for both MHCI and MHCII-DRB (Additional file 1: Tables S8-S13). TSP across such large divergence times are unlikely due only to neutral processes and are thus more likely the consequence of long-term balancing selection or convergent evolution (i.e., balanced TSP). However, in the case of recently diverged species it is more parsimoniously explained by incomplete lineage sorting during speciation and/or posterior introgression events (i.e., neutral TSP) [52]. Both processes have been described for the *Lynx* genus. A discordant phylogenetic pattern for mitochondrial DNA was found whereby *L. canadensis* appeared as *L. pardinus* sister species, a signal of incomplete lineage sorting, and pervasive signals of introgression between the *L. canadensis* and *L. lynx* and between *L. pardinus* and *L. lynx* have been reported [34, 35]. A more detailed study, taking into account broader genomic regions and ideally MHC alleles assigned to loci, would be required to tackle whether the origin of each trans-species polymorphism in lynx is due to introgression, incomplete lineage sorting, convergent evolution or mere lack of genetic divergence caused by recent species divergence times.

### Evidence of natural selection

Both MHCI and MHCII-DRB alleles showed signals of purifying selection at non-ABS, stronger in MHCII-DRB than in MHCI. However, we did not find signatures of long-term positive selection in the form of an increased d_N_/d_S_ ratio at ABS for any MHC family. Contrastingly, such positive selection signals were previously described in analyses of MHCI in cheetah [30], bengal tiger [32] and leopard [31], and for MHCII-DRB in eight different Felidae lineages [28], cheetah [30], but not in bengal tiger [32], leopard [31] or Eurasian lynx [29]. Interestingly, for MHCI we found d_S_ at the ABS to be much larger than d_S_ at non-ABS, this indicates that the ABS regions are older than non-ABS and points to positive selection acting in presence of recurrent gene conversion events [45].

In the site-by-site selection test, the amino acids found to be under positive selection in each class had previously been reported in felids (i.e., the 77th amino acid of the MHCI was described as one of the highly polymorphic positions located at the α_1_ by Yuhki et al. [24, 25] and the 86th amino acid of the MHCII-DRB was found to be under positive selection by Wei et al. [28] when comparing different Felidae species). It must be noted that the pooling of alleles across loci will generally reduce the power of selection detection tests, so ours and others results might be too conservative, but not prone to false positives [51]. The fact that we don’t find the Iberian lynx to be enriched for pseudogenes also suggests that the relaxation of purifying selection observed across the genome has not affected MHC variation in this way [35].

### Genetic drift and balancing selection

One of the main objectives of this study was assessing the possible role of balancing selection in maintaining genetic diversity in declining species by comparing allele repertoires in the two sister lynx species with contrasting demographic histories and overall genetic diversity. We can use the combination of alleles observed in Iberian and Eurasian lynx as a proxy for the ancestral variation in the Iberian lynx. This seems reasonable given the recent divergence and posterior gene flow, and is somehow supported by the nearly complete overlap of allele repertoires between these two species (also between these and Canada lynx) (Fig. 3). Loss of diversity at MHC in bottlenecked populations is a common observation, and suggests that genetic drift is able to override any ongoing balancing selection in most scenarios [19–22]. However, the retention of allelic and nucleotide diversity at MHC exons is quite striking given the extreme overall genetic erosion reported in the Iberian lynx genome, with over 80% of the coding sequences showing no variation at all and overall SNP density being at most one third of that of Eurasian lynx [35]. Furthermore, despite the loss of allelic diversity, Iberian lynx conserved a similar number of segregating sites and maintained high average levels of divergence between alleles, with most supertypes being represented by at least one allele. In fact, the retention of variation- especially of a non-random highly-divergent subset of alleles- following a bottleneck has been observed in several other studies (e.g., [53–56]). In these cases, this has been interpreted as the consequence of balancing selection favouring the retention of more divergent vs. less divergent alleles (divergent allele selection [14]).

An often overlooked fact is that MHC are multigene families with rather complex evolution, and the outcome of increased drift imposed by a bottleneck could depend on the evolutionary relationships of alleles across paralogs. In the absence of gene conversion, which shuffles and homogenizes alleles both within and across loci, the duplicated gene copies would evolve reciprocally monophyletic alleles sets, so that the action of drift would necessarily result in the retention of divergent alleles even if all copies become fixed for one allele, as at least one representative of each divergent lineage would be kept in each locus. It is thus not parsimonious to invoke balancing selection without first discarding this pre-existing pattern (as suggested by Van Oosterhout [57] for Ellison et al. data [58]). Although, as most other studies in non-model species, we could not attribute alleles to particular loci, several lines of evidence suggest that gene-conversion and recombination are pervasive in the MHC region, preventing the formation of reciprocal monophyly across loci. First, in domestic cat allelic lineages do not correspond to loci, as all alleles belonged to the same phylogenetic lineage in some individuals [26]. Second, in humans MHCI introns have been homogenized relative to exons, as expected when gene conversion is present but balancing selection operates to maintain diversity in the latter but not in the former [59]. Third, the “patchwork pattern” found in the MHC exons is explained by gene conversion mediated minor segmental exchange [24, 25, 60, 61]. Fourth, the highly elevated d_S.ABS_/d_S.non-ABS_ ratio found here at MHCI in lynx and elsewhere in other species [30] indicates that gene-conversion occurred recurrently in evolutionary times, at least following locus duplication [45].

If recombination and gene-conversion events indeed have shaped the lynxes MHC region up to a point in which allelic lineages are no longer monophyletic by locus, the retention of divergent alleles may be taken as evidence to support the action of balancing selection. Balancing selection could be operating within and also between loci, allowing the extension of the target of balancing selection to mutilocus MHC haplotypes [14, 62]. In this regard, MHC haplotypes (i.e., allele combinations at several tightly linked loci) could thus be selected to harbour a set of highly divergent (and thus functionally complementary) alleles giving the individuals with such haplotypes a “permanent heterozygote” advantage [14, 62–64]. Such process will be especially relevant for small or declining populations where due to low within locus allelic diversity and heterozygosity, interlocus diversity will become the main source of intraindividual functional variation.

Disentangling which mechanisms are the main drivers of the retention of functional variation in the Iberian lynx will require data at the population and individual levels, as well as locus-specific genotypes. Such study would expand our empirical knowledge on how the evolutionary forces shape the genomic landscape of endangered species.

### Generation of novel functional variation

The Iberian lynx allelic repertoire was mostly a subset of the Eurasian lynx alleles. However, it included a pair of Iberian-private alleles (Fig. 3). One of these private alleles (Lypa-DRB*6) appears to have been originated by recombination of alleles within the species repertoire while the other (Lypa-DRB*7) derived from it and contained a single non-synonymous point mutation. Although we cannot discard the possibility of these alleles being ancestral to both species and being lost in Eurasian, an alternative explanation is that it was originated within the Iberian lynx lineage. In fact, these two alleles define a novel and exclusive lineage and supertype, not represented in any of the other felids analysed. Indeed, recombination and gene conversion have long been described as a motor of rapid evolution compared to point mutation in multigene families [65, 66], with special mention to the MHC families [45, 67]. Short exonic fragments passed to different alleles by gene-conversion or interchanged by reciprocal recombination events would increase the number of alleles faster compared to point mutation acting alone, especially in the presence of interlocus recombination [67]. Only recently, such processes have been invoked as a process by which genetically depauperated populations could recover some of the variability lost through genetic erosion [68]. Additionally, the non-synonymous point mutation that created Lypa-DRB*7 from Lypa-DRB*6 originated a similar but functionally distinct allele, suggesting that mutation did also contribute, although to a lesser extent, to create new functional variation (Fig. 3, Additional file 1: Tables S2 and S13). This amino acid change is at a non-ABS site though, so we cannot gauge whether it has increased its frequency because of positive selection or instead by the relaxation of purifying selection. Non-ABS sites have clear signals of long-term purifying selection (Table 1), but the Iberian lynx genome contains pervasive signals of recent relaxation, as reflected in excess of non-synonymous variants and substitutions [35]. The fact that these new alleles were able to attain allelic frequencies high enough to persist in the species despite the series of demographic bottlenecks it has suffered since its speciation [35] points to some form of positive or balancing selection [13, 14].

### Iberian lynx immunity and population viability

Irrespective of the evolutionary mechanisms involved, we showed retention of substantial functional variation at MHC despite severe overall genomic erosion in the Iberian lynx. These are good news for the conservation of Iberian lynx. Notwithstanding, several studies suggest that the immune system of the Iberian lynx is compromised and have alerted on its consequences for population viability. A recent increase in disease-associated mortality rates for the Doñana population and the species as a whole has been reported [69, 70]. In particular, a feline leukaemia virus (FeLV) outbreak in 2007 killed more than half of the twelve infected animals in less than six months, leaving the main reproductive nucleus in Doñana without males [71–73]. A latter study confirmed that the strain responsible of the outbreak was not specially virulent [74], as it did not exhibit genetic variants known to confer increased virulence and it did not severely affect cats in a in vivo transmission study, highlighting the special susceptibility of Iberian lynxes to infectious diseases. Based on that apparent deficiency of immunity in Iberian lynxes and the frequent detection of infectious agents among sympatric carnivores [75], a disease outbreak has been considered a serious risk for Iberian lynx persistence [71, 75, 76].

We should not forget that the diversity measures presented in this study are referred to the species level and may not properly reflect the diversity at population or individual levels, which are the levels at which natural selection act. If the repertoire of alleles of a species is sorted out in different sets corresponding to distinct populations, the levels of each population’s diversity will be low- even when the species diversity is high. Likewise, if the allelic frequencies within a population are very skewed, the levels of individual variation will be low- even when the population allelic richness is high. Hence, we need to extend our characterization of MHC variation to Iberian lynx populations and individuals before we can fully evaluate its impact on immune function and ultimately on disease susceptibility and population viability. Furthermore, the immune response is a complex physiological process involving many genes, among which MHC are an important but limited part [77]. Thus, a more robust evaluation of immunocompetence based on genetic information would require the assessment of the whole immunome.

## Conclusions

The fate of functional variation following a bottleneck could be to follow the neutral variation dynamics towards depletion, or to be, at least partially, maintained by balancing selection. Based on MHC data from the *Lynx* genus, we argue that functional genetic variation can be retained even in the face of extreme genetic erosion through the functional redundancy provided by multigene families and the possible contribution of different forms of balancing selection. If generalized to other species and functional components, especially those based on multigene families, it would mean that endangered species might retain more adaptive capacity than we would predict from diversity at neutral markers.

## Methods

### Sampling

Our sampling covered the whole distribution of *L. pardinus* and a remarkable part of the distribution of *L. lynx*. We used 102 samples of the two remnant populations of Iberian lynxes (Doñana(*N* = 48) and Sierra Morena(*N* = 54)), and 99 samples of six populations of Eurasian lynxes (Estonia (*N* = 15), Latvia (*N* = 15), Norway (*N* = 11), Poland(*N* = 20) Russia-Kirov (*N* = 14), and Russia-Vladivostok (*N* = 24)) (Fig. 1). Admittedly, some of these Eurasian lynx populations went through bottlenecks and show reduced genetic diversity [39] but this will not bias our species level estimates. Given that our aim here is to get the most complete allelic repertoire for each species, the inclusion of bottlenecked populations can only add some new alleles or not, but will not artificially reduce the allelic repertoire of the species. *L. canadensis* and *L. rufus* are represented for comparative purposes by two individuals per species.

### Primer design and amplification strategy

Two sets of primers were designed to amplify the second exon of both MHC class I and MHC class II-DRB genes. Primer design used for MHCI amplification is described elsewhere [49] and MHCII-DRB design followed the same rationale. In short, primer design was based on a set of variants that included all MHC class I or class II-DRB exon 2 alleles for species closely-related to lynxes available in GenBank as of May of 2011 (i.e., all Felidae alleles.) and the annotated region of the cat [27]. Also the transcripts from two different Iberian lynxes annotated as MHCI or MHCII-DRB by the Iberian lynx genome project [35] were included. Primers were designed in approximately the same regions as the ones used in previous studies on MHC variation in the Felidae to enable comparisons [28, 30–32]. More specifically, MHCI primers spanned bases 2 to 21 (forward) and 253 to 271 (reverse) of the human MHC class I exon 2 and MHC class II-DRB spanned bases 4 to 23 (forward) and 270 to 288 (reverse) of the human homologues [78–80].

For MHCI we used a PCR amplification strategy we called Pooled-primers designed to maximize the number of detected alleles by minimizing amplification bias among alleles [49]. On the contrary, given that the MHCII-DRB required less degenerated primers, we used a standard PCR using a single pair of degenerate primers: Fel_MhcII_ex2_F [5′-TGTBYCCACAGCACATTTCY-3′] and Fel_MhcII_ex2_R [5′-CTCAMCTCGCCGSTGCAC-3′].

### Amplification and sequencing of MHC loci

Genomic DNA was extracted from blood or tissue using standard phenol-chloroform methods [81]. To produce sequencing libraries, we used the Universal Tailed Amplicon Sequencing design by Roche consisting of a two-round PCR approach in which the first round amplifies the target loci using primers with a universal 5′ extension and the second adds 454 sequencing adapters and a sequence tag to the amplicons generated in the first PCR to unambiguously identify each sample. Artefact formation during PCR was minimized by using the Phusion High-Fidelity PCR Kit by Roche, which reduces nucleotide misincorporation [82], and by implementing long extension times and no final extension step, which prevents chimera formation. PCRs were run in a final volume of 10 μl following manufacturer indications. Cycling conditions were the same for all PCRs: an initial denaturation at 98° for 30 s and 25 cycles of 98 °C for 10 s, 57 °C for 30 s and 72 °C for 2 min. PCR products were equimolarly pooled and sequenced on a 454 GS Junior system.

### Data processing and allele validation

High throughput reads were sorted by individual, quality filtered, collapsed into unique sequences and assigned to alleles or artefacts following Sommer et al. 2013 (refer to [83] for further details). This protocol assumes different amplification efficiencies for different alleles and does not assume that reads representing true alleles are more abundant in the read pool than any artefact. These two aspects are important when primers are not guaranteed to amplify alleles with the same efficiency. The sole modification introduced to the method was that we did not systematically replicate each individual in the case of *L. lynx* and *L. pardinus*. Due to the low genetic variation of the species [35, 42], and the use of several individuals belonging to the same population, we expected every allele to be found in more than one individual. Nevertheless, a minimum of 10% of the samples were replicated as quality control. Alleles were named following Klein et al. [84]. To be considered as functional and taken into account in downstream analysis an allele had to lack signs of pseudogenization (i.e., they should not present indels, reading frame shifts or premature stop codons). All Iberian lynx alleles without pseudogenization signals, were confirmed to be transcribed by RNA-Seq or RT-PCR amplicon (data not shown); no similar data was available for non-Iberian lynx alleles.

### Data analysis

#### Recombination and phylogenetic relationships of the alleles

Single Break Point (SBP) and Genetic Algorithm Recombination Detection (GARD) tests [85] were used to identify recombination breakpoints. Such breakpoints and the phylogenetic trees produced as output were used to inform subsequent site-by-site selection tests. To evaluate relationships among sequences, we constructed a phylogenetic network using SplitsTree4 [86, 87]. We used the Neighbor-Net method and Jukes-Cantor distances. Contrary to traditional phylogenetic trees, phylogenetic networks enable visualization of conflicting signals from processes such as gene conversion and recombination [87].

Alleles found in the *Lynx* genus were compared among them and to all the Felidae alleles already available in GenBank to detect instances of functional variation shared across the genus and across the Felidae family. For this purpose we first looked for shared alleles at both nucleotide and amino acid levels. Even though phylogenetic trees are inappropriate to represent phylogenetic relationships of alleles that suffered recombination or gene-conversion, we used them to explore how lynx alleles are related to others in the Felidae family. For MHCII-DRB, for which previous allele lineages have been described [26, 28], we recorded which of those lineages are represented in the *Lynx* genus. Trees were constructed in the Geneious suite [88] using RAxML [89] under the GTR GAMMA I nucleotide model and 1000 bootstraps. Consensus tree was calculated using the suite’s consensus tree builder tool.

The *Lynx* genus showed shared polymorphisms also with other felids, as we expected both alleles phylogenetic trees and the supertypes clustering show the alleles of different species clustering together rather than clustering them by species (Additional file 2: Figures S3-S6). We even found lynx alleles to be completely identical at the nucleotide and/or amino acid level to alleles present in other felid species. For the MHCI, one allele was shared between *L. rufus* and *F. catus* at the nucleotide level (*FLAI-O* = Lyru-MHCI*11, see Additional file 1: Table S8), while two sets of alleles were shared at the protein level: one including all the lynxes, cat and tiger and the second one with all the lynxes and cat only (Additional file 1: Table S9). At the supertype level, 12 supertypes contained lynx alleles and 11 of them also contained alleles from other felid species (Additional file 1: Table S10). No MHCII-DRB allele was shared between lynxes and the rest of the felids at a nucleotide level (Additional file 1: Table S11) and only one pair was shared between *Lynx lynx* and *Neofelis nebulosa* at the protein level (Nene-DRB*202 = Lyly-DRB*09, Additional file 1: Table S12). Such alleles shared across distant species and showing no or very low divergence, pose a typical example of TSP and are likely to have been subject of balancing selection. When compared to the five MHCII-DRB lineages described by Yuhki and O’Brien [26] we found most lynx alleles to cluster in lineages 2 and 5, as found in Wei et al. [28] for *L. lynx* alleles (Additional file 2: Figure S4). Likewise, we found no lynx alleles in lineages 4 and 1. However, we did not find many lynx alleles on an uncertain lineage, as reported by Wei et al. [28], but those alleles grouped instead in lineage 5, and Pati-DRB*1 (which was the only non-lynx allele on such uncertain clade) on the base of the tree. Additionally, we found a bobcat allele clustering in lineage 3, where no lynx alleles had been described before.

#### Signatures of selection

Antigen binding sites (ABS) codon positions were inferred them from human MHC class I [79, 80] and from MHCII-DRB [78] homologs. Additionally, we checked that the positions attributed to the ABS were equivalent to those reported in other felids [30, 31]. Positive and purifying selection at putative ABS and non-ABS codons were inferred by an unbiased estimation of the rates of nonsynonymous (d_N_) and synonymous (d_S_) substitution [90], calculated by the *kaks* function in the *Seqinr* R package [91]. The significance of the deviation above or below zero of the d_N_/d_S_ ratio was calculated using a Mann-Whitney U-test.

To infer site-by-site positive and negative selection in our alleles, we ran the four tests available at *datamonkey* web server [92] (i.e., SLAC,REL, FEL [46] and FURBAR [93]. For all analyses, we used the model of evolution that best fitted the data and the recombination information when the alignment spanned recombination breakpoints. Finally, we used the Integrative Selection Results tool to compare the results.

#### Functional diversity retention: Genetic diversity and supertypes

All analysis were performed for the whole dataset of alleles found across species as well as for each species on its own using different R packages. We used *ape* [94] to calculate the number of segregating sites as well as the number of nucleotide differences and *pegas* [95] to calculate nucleotide diversity. Nucleotide genetic distances were estimated using *ape* while *Phangorn* [96] was used to estimate amino acid distances for the whole sequence, for ABS and for non-ABS. The evolutionary model to be used in each case was inferred using *Model Selection* package implemented in the web server hosted at http://www.datamonkey.org [92].

Second, to evaluate the retention of functional variation beyond TSP, we grouped alleles into supertypes based on functional similarity [97]. We used a pocket-based approach in which only the ABS are taken into account to group the alleles. Each ABS amino acid was transcribed into five physicochemical relevant descriptor variables: z1 (hydrophobicity), z2 (steric bulk), z3 (polarity), z4 and z5 (electronic effects) [98]. Later, hierarchical clustering with euclidean distance was applied to the resulting allele matrix using dist and hclust functions from the stats R package to group alleles based on their functional (antigen-binding) properties.

## Additional files

Additional file 1: Table S1. Nucleotide sequence alignment of the second exon of MHC class I alleles observed in Lynx. Numbers indicate the nucleotide positions inferred from human MHC class I loci (Bjorkman et al. 1987; Bjorkman & Parham 1990). Dots indicate identity to allele Lypa_MHCI*10/Lyly_MHCI*10, which is used as reference. **Table S2.** Nucleotide sequence alignment of the second exon of MHC class II-DRB alleles observed in Lynx. Alleles are aligned to Lypa_DRB*1/Lyly_DRB*1/Lyca_DRB*1/Lyru_DRB*1. Numbers indicate the nucleotide positions of exon 2 inferred from the human sequence (Brown et al. 1993). Dots indicate identity to the top sequence. **Table S3.** Lynx MHCI pseudogenization signatures. For each allele we show whether it contains insertion and/or deletions (along with the inserted base and its position), whether it shows an Open Reading Frame (ORF), and if it was considered as a pseudogene in this study. **Table S4.** Integrative MHCI positive and negative selection tests. dN-dS ratios for codon showing signals of selection in any of the test performed (SLAC, FEL, REL, and FURBAR) are reported along with their significance values (either *p*-value or Bayes factor), whether they were significant for each test (+) or not (−), and if they were considered in this study (Yes/No), with those considered also shaded in light blue. **Table S5** Amino acid sequence alignment of the second exon of MHC class I alleles observed in Lynx. Alleles are aligned to Lypa_DRB*10/Lyly_DRB*10. Numbers indicate the amino acid positions and asterisks putative ABS inferred from human MHC class I loci (Bjorkman et al. 1987; Bjorkman & Parham 1990). Dots indicate identity to the top sequence. The amino acids inferred to be under positive or negative selection are marked with a + or a – sign, respectively. **Table S6.** Integrative MHCII-DRB positive and negative selection tests. dN-dS ratios for codon showing signals of selection in any of the test performed (SLAC, FEL, REL, and FURBAR) are reported along with their significance values (either *p*-value or Bayes factor), whether they were significant for each test (+) or not (−), and if they were considered in this study (Yes/No), with those considered also shaded in light blue. **Table S7.** Amino acid sequence alignment of the second exon of MHC class II-DRB alleles observed in lynxes. Alleles are aligned to Lypa_DRB*1/Lyly_DRB*1/Lyca_DRB*1/Lyru_DRB*1. Numbers indicate the amino acid positions and asterisks putative ABS inferred from human MHC class I loci (Brown et al. 1993). Dots indicate identity to the top sequence. The amino acids inferred to be under positive or negative selection are marked with a + or a – sign, respectively. **Table S8.** Unique MHCI nucleotide alleles found across Felidae. Each Felidae allele were collapsed into unique nucleotide sequences and the instances of identical sequences being shared by different species were identified. For each shared sequence we show an identification number (Seq), the number of species in which it appears (#Species), the acromin of such species (Species), whether it is present in any Lynx species (Lynx), whether it is shared by any Lynx species and one non-Lynx species (Lynx_Other), the number of alleles collapsed (#Alleles), and the alleles names (Alleles names). **Table S9.** Unique MHCI amino acid alleles found across Felidae. Each Felidae allele were collapsed into unique amino acids sequences and the instances of identical sequences being shared by different species were identified. For each shared sequence we show an identification number (Seq), the number of species in which it appears (#Species), the acromin of such species (Species), whether it is present in any Lynx species (Lynx), whether it is shared by any Lynx species and one non-Lynx species (Lynx_Other), the number of alleles collapsed (#Alleles), and the alleles names (Alleles names). **Table S10.** Felidae MHCI supertype definition based on hierarchical clustering. Each Felidae allele was clustered following a pocket based approach that considered only ABS. For each allele we show its name (Allele) and the supertype it was assigned to within the Felidae analysis(Supertype). *Lynx* alleles are shown in bold. For each supertype we report whether it contains at least one *Lynx* allele, and if so we indicate the supertype cluster they correspond to in Fig. 3a (Supertype present in *Lynx* genus) and whether this was exclusive to *Lynx* or shared with other felid species (Supertype shared with other felids). **Table S11.** Unique MHCII-DRB nucleotide alleles found across Felidae. Each Felidae allele were collapsed into unique nucleotide sequences and the instances of identical sequences being shared by different species were identified. For each shared sequence we show an identification number (Seq), the number of species in which it appears (#Species), the acromin of such species (Species), whether it is present in any *Lynx* species (Lynx), whether it is shared by any *Lynx* species and one non-*Lynx* species (Lynx_Other), the number of alleles collapsed (#Alleles), and the alleles names (Alleles names). **Table S12.** Unique MHCII-DRB amino acid alleles found across Felidae. Each Felidae allele were collapsed into unique amino acids sequences and the instances of identical sequences being shared by different species were identified. For each shared sequence we show an identification number (Seq), the number of species in which it appears (#Species), the acromin of such species (Species), whether it is present in any *Lynx* species (Lynx), whether it is shared by any *Lynx* species and one non-Lynx species (Lynx_Other), the number of alleles collapsed (#Alleles), and the alleles names (Alleles names). **Table S13.** Felidae MHCII-DRB supertype definition based on hierarchical clustering. Each Felidae allele was clustered following a pocket based approach that consireded only ABS. For each allele we show its name (Allele), the supertype it was assigned to (Supertype). *Lynx* alleles are shown in bold. For each supertype we report whether it contains at least one *Lynx* allele, and if so we indicate the supertype cluster they correspond to in Fig. 3a (Supertype present in *Lynx* genus) and whether this was exclusive to *Lynx* or shared with other felid species (Supertype shared with other felids). (XLS 350 kb)

Additional file 2: Figure S1. Phylogenetic network of Lynx MHCI alleles. We constructed a network with the Neighbor-Net method and Jukes-Cantor distances in SplitsTree4. Alleles are colored by species of origin. **Figure S2.** Phylogenetic network of Lynx MHCII-DRB alleles. We constructed a network with the Neighbor-Net method and Jukes-Cantor distances in SplitsTree4. Alleles are colored by species of origin. **Figure S3.** Phylogenetic tree of Felidae MHCI alleles. The tree was constructed with RaxML. Tips are colored by genus of origin. **Figure S4.** Phylogenetic tree of Felidae MHCII_DRB alleles. The tree was constructed with RaxML. Tips are colored by genus of origin. **Figure S5.** Felidae MHCI supertype definition. We used average hierarchical clustering and a cut-off threshold of Euclidean distance ≥15 to classify alleles into distinctive functional groups (supertypes, red boxes) based on functional similarity at their ABS. Alleles with identical amino acids at ABS are represented as tip polytomies at 0 Euclidean distance. **Figure S6.** Felidae MHCII-DRB supertype definition. We used average hierarchical clustering and a cut-off threshold of Euclidean distance ≥15 to classify alleles into distinctive functional groups (supertypes, red boxes) based on functional similarity at their ABS. Alleles with identical amino acids at ABS are represented as polytomies at 0 euclidean distance. (PDF 408 kb)
