# Supplementary material for: Retention of functional variation despite extreme genomic erosion: MHC allelic repertoires in the Lynx genus
Source: BMC Evol Biol. 2017 Jul 4;17:158. doi: 10.1186/s12862-017-1006-z (PMC5496644; doi:10.1186/s12862-017-1006-z)
Supplement: Supplementary file 2 — Phylogenetic network of Lynx MHCI alleles. We constructed a network with the Neighbor-Net method and Jukes-Cantor distances in SplitsTree4. Alleles are colored by species of origin. Figure S2. Phylogenetic network of Lynx MHCII-DRB alleles. We constructed a network with the Neighbor-Net method and Jukes-Cantor distances in SplitsTree4. Alleles are colored by species of origin. Figure S3. Phylogenetic tree of Felidae MHCI alleles. The tree was constructed with RaxML. Tips are colored by genus of origin. Figure S4. Phylogenetic tree of Felidae MHCII_DRB alleles. The tree was constructed with RaxML. Tips are colored by genus of origin. Figure S5. Felidae MHCI supertype definition. We used average hierarchical clustering and a cut-off threshold of Euclidean distance ≥15 to classify alleles into distinctive functional groups (supertypes, red boxes) based on functional similarity at their ABS. Alleles with identical amino acids at ABS are represented as tip polytomies at 0 Euclidean distance. Figure S6. Felidae MHCII-DRB supertype definition. We used average hierarchical clustering and a cut-off threshold of Euclidean distance ≥15 to classify alleles into distinctive functional groups (supertypes, red boxes) based on functional similarity at their ABS. Alleles with identical amino acids at ABS are represented as polytomies at 0 euclidean distance. (PDF 408 kb) [file 12862_2017_1006_MOESM2_ESM.pdf]

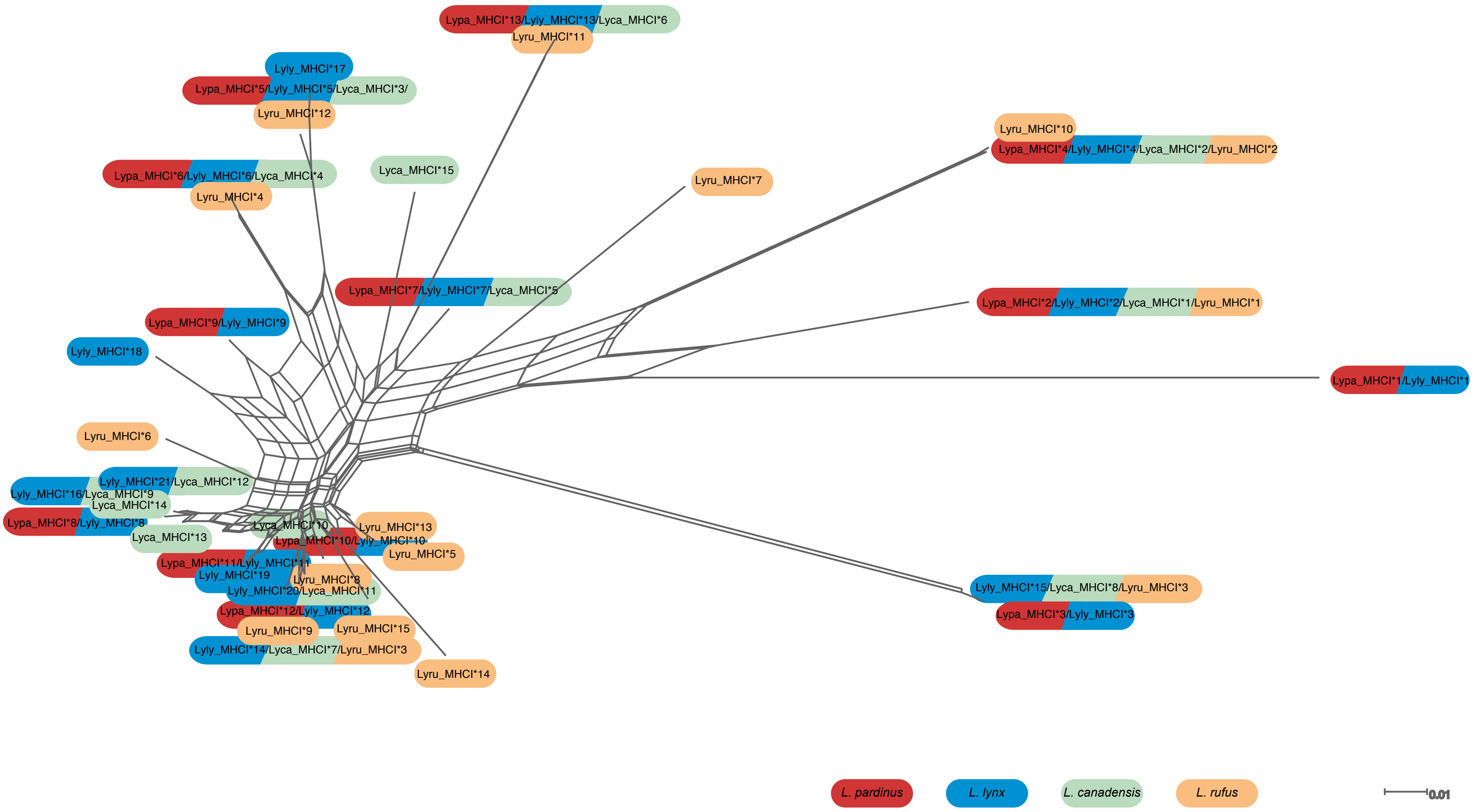

Figure S1. Phylogenetic network of Lynx MHCI alleles. We constructed a network with the Neighbor-Net method and Jukes-Cantor distances in SplitsTree4. Alleles are colored by species of origin.

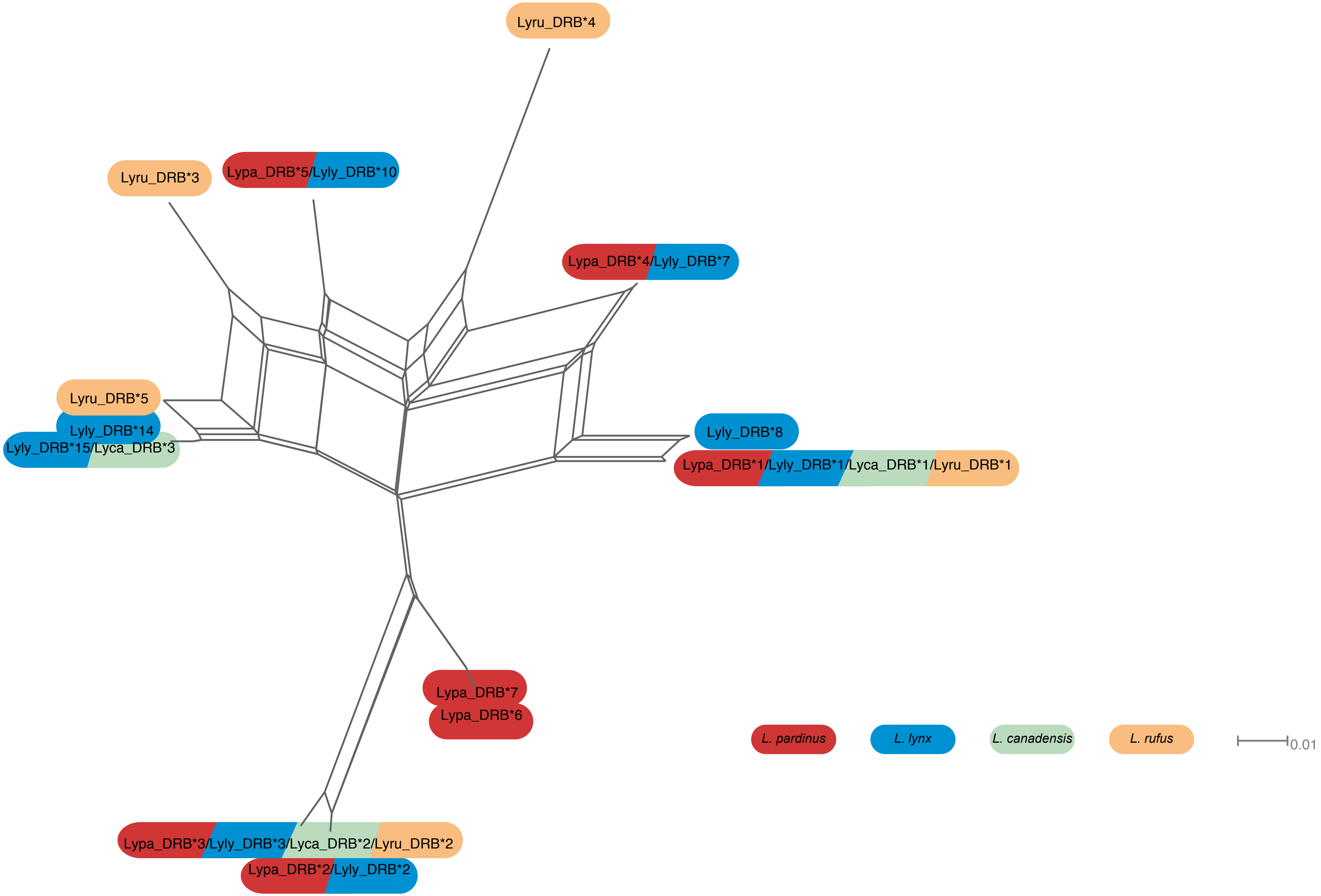

Figure S2. Phylogenetic network of Lynx MHCII-DRB alleles. We constructed a network with the Neighbor-Net method and Jukes-Cantor distances in SplitsTree4. Alleles are colored by species of origin.

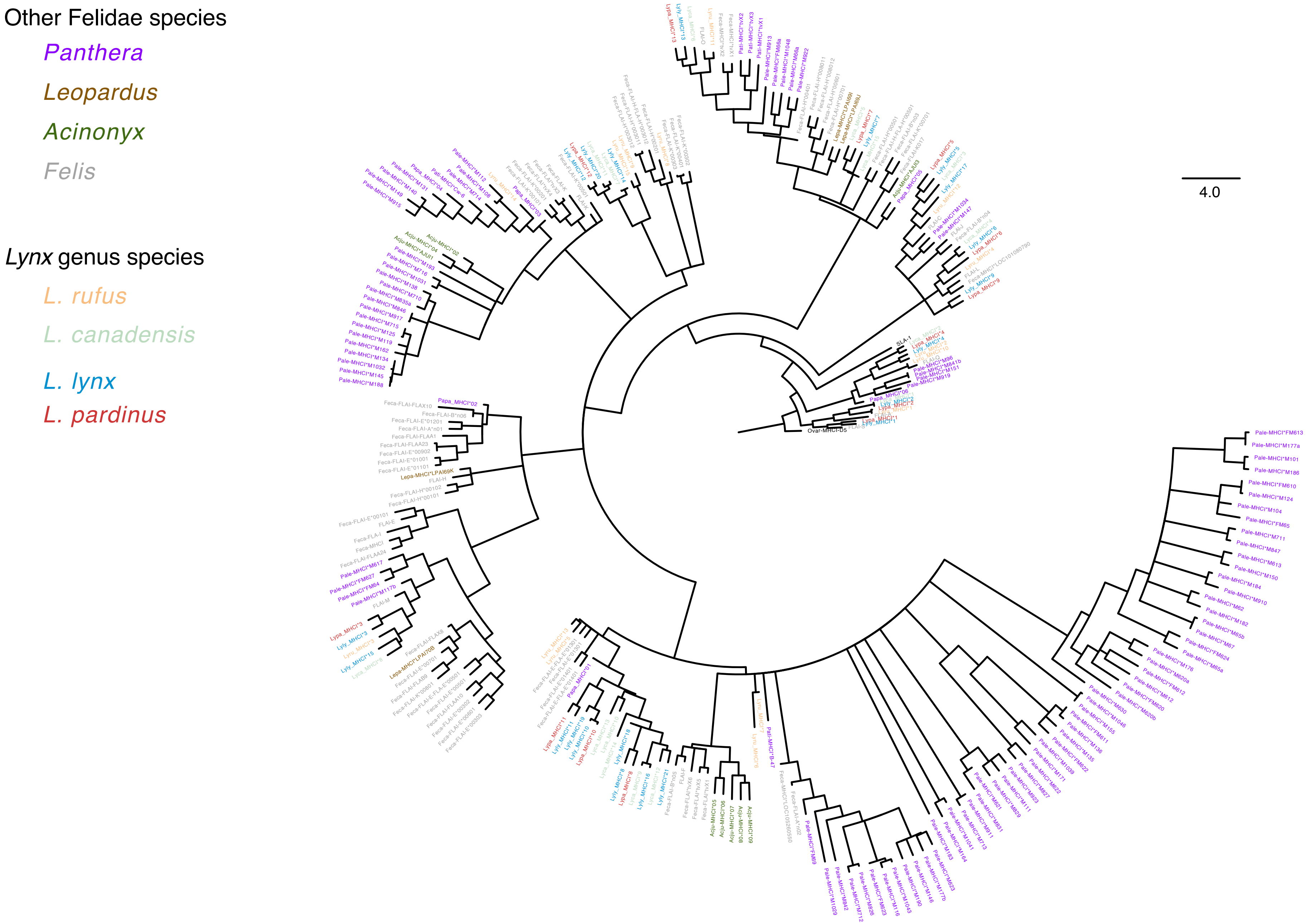

Figure S3. Phylogenetic tree of Felidae MHCI alleles. The tree was constructed with RaxML. Tips are colored by genus of origin.

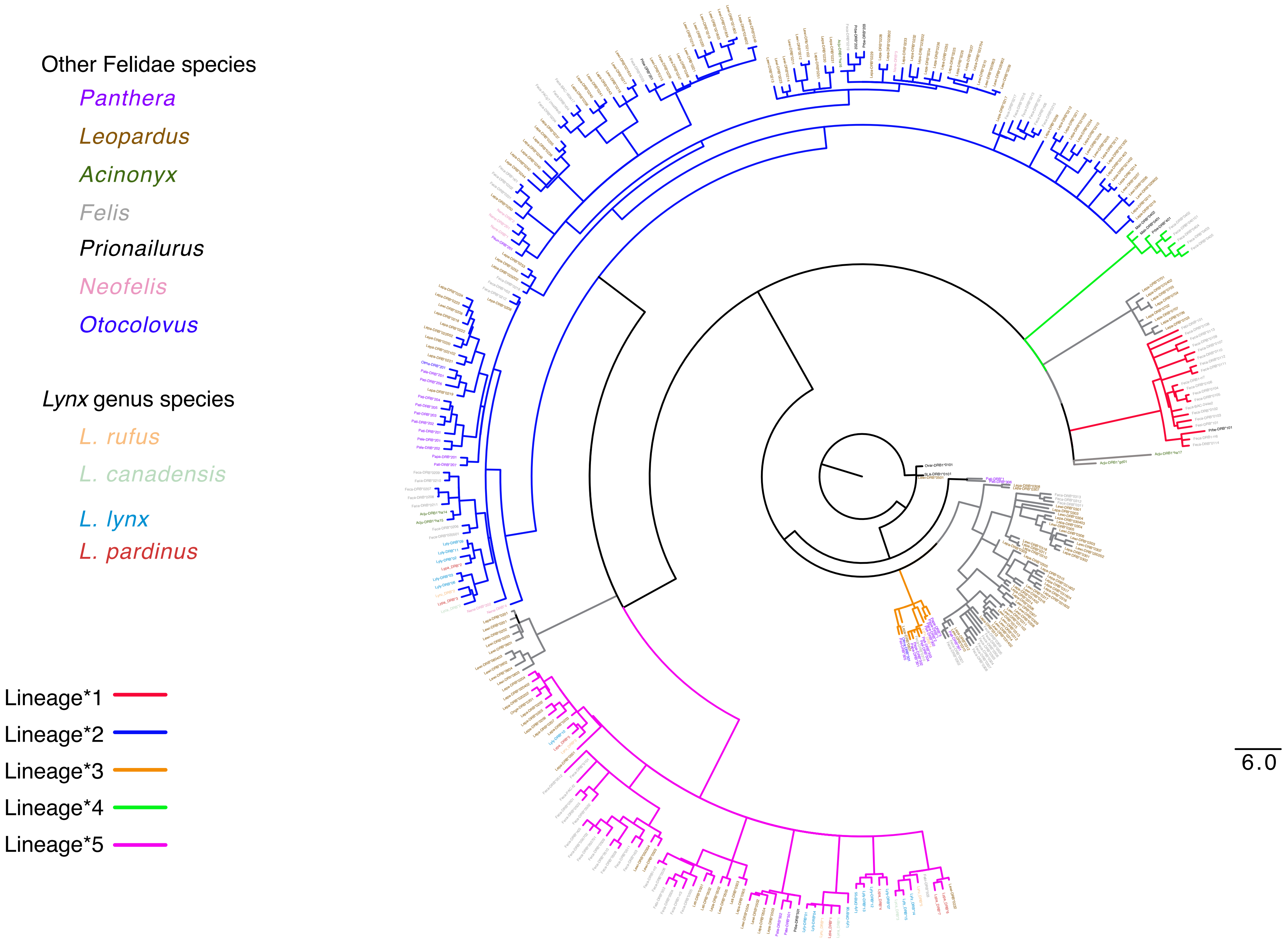

Figure S4. Phylogenetic tree of Felidae MHCII_DRB alleles. The tree was constructed with RaxML. Tips are colored by genus of origin.

1. Yuhki N, OBrien SJ: Nature and origin of polymorphism in feline MHC class II DRA and DRB genes. J Immunol 1997, 158(6):2822-2833.
2. Wei K, Zhang ZH, Wang XF, Zhang WP, Xu X, Shen FJ, Yue BS: Lineage pattern, trans-species polymorphism, and selection pressure among the major lineages of feline Mhc-DRB peptide-binding region. Immunogenetics 2010, 62(5):307-317.

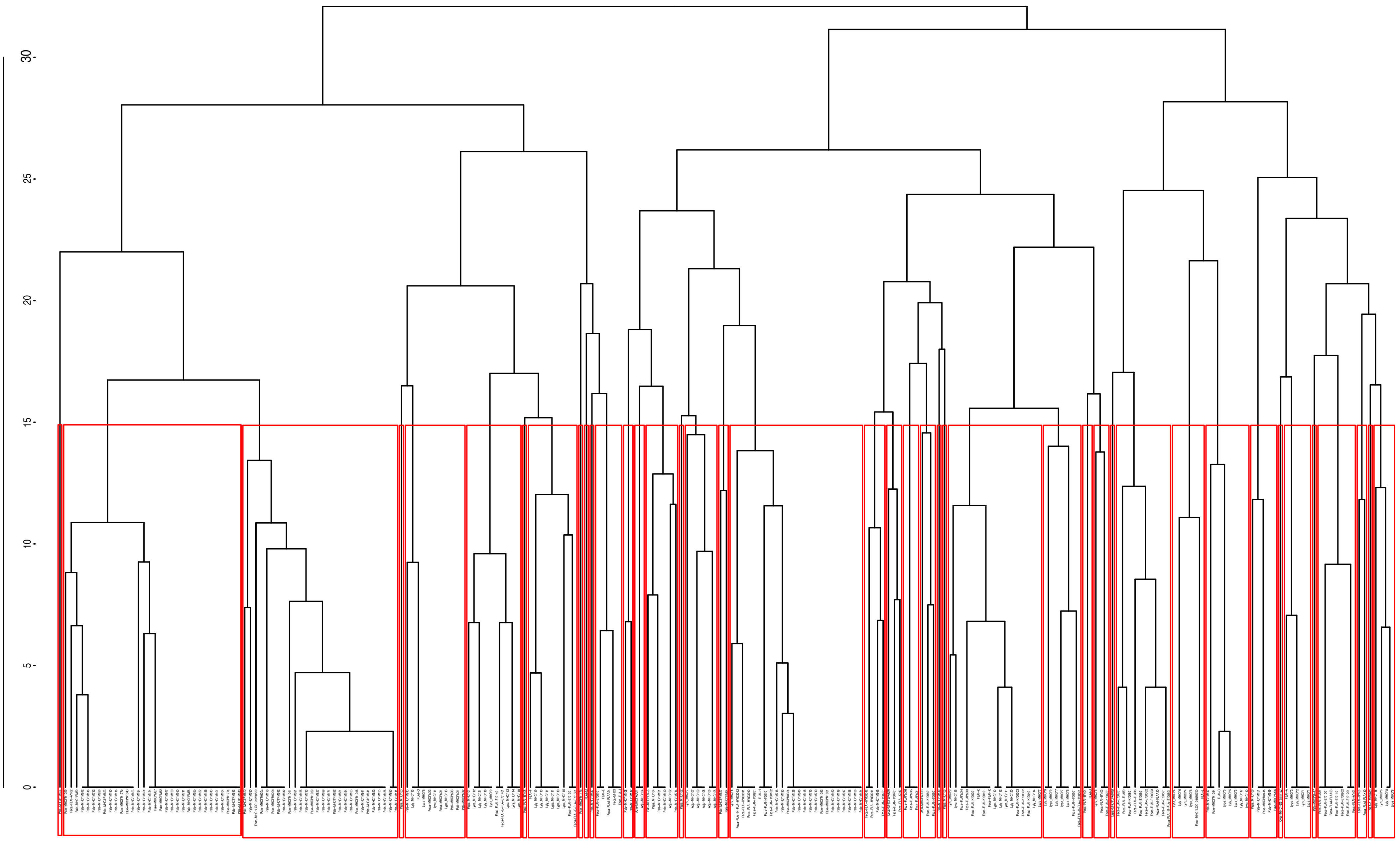

Figure S5. Felidae MHCI supertype definition. We used average hierarchical clustering and a cut-off threshold of Euclidean distance ≥15 to classify alleles into distinctive functional groups (supertypes, red boxes) based on functional similarity at their ABS. Alleles with identical amino acids at ABS are represented as tip polytomies at 0 Euclidean distance.

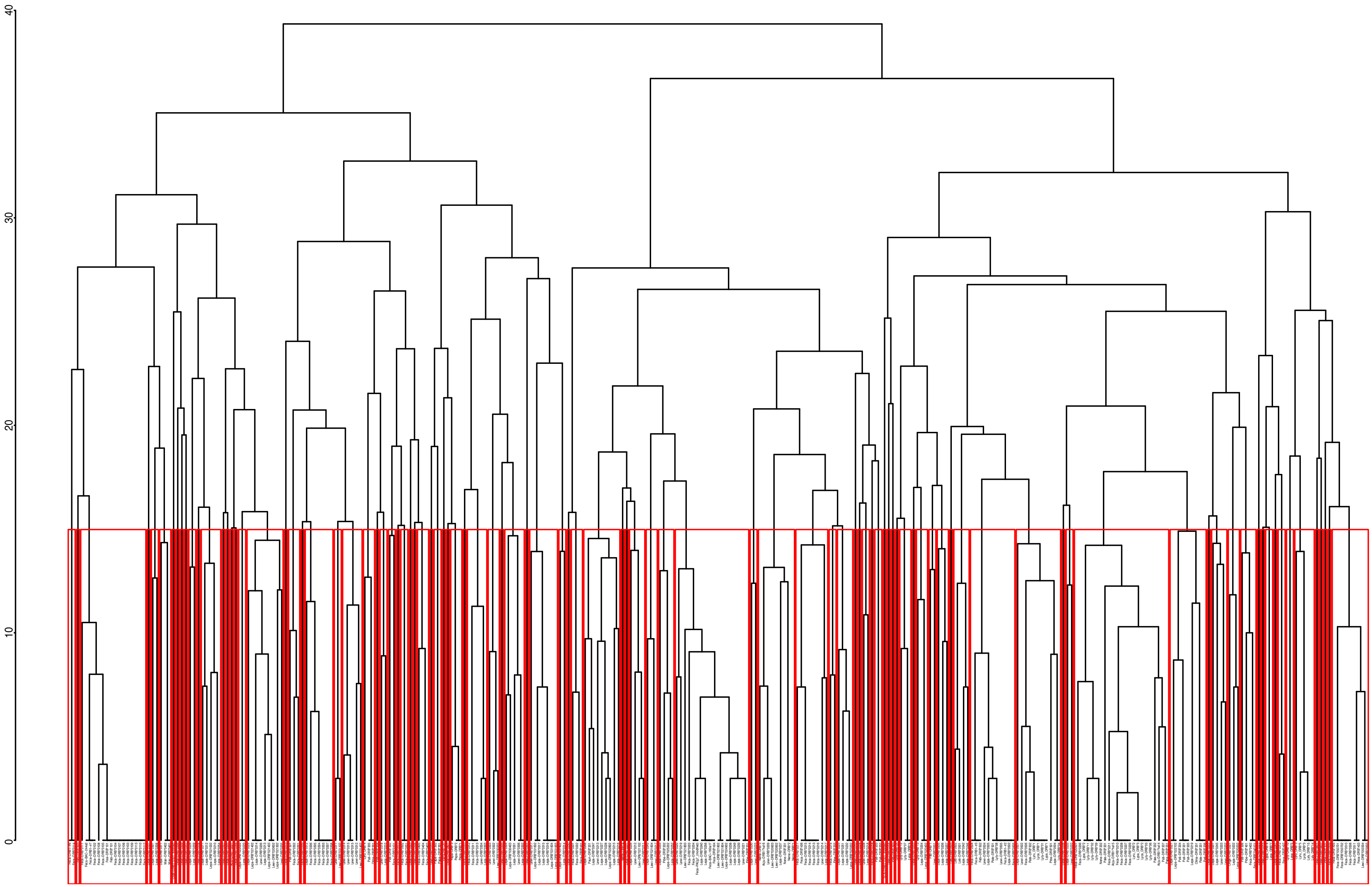

Figure S6. Felidae MHCII-DRB supertype definition. We used average hierarchical clustering and a cut-off threshold of Euclidean distance ≥ 15 to classify alleles into distinctive functional groups (supertypes, red boxes) based on functional similarity at their ABS. Alleles with identical amino acids at ABS are represented as polytomies at 0 euclidean distance.
